# Supplementary material for: Perceived attitudes toward LGBTQ+ physicians among Thai patients with psychiatric disorder: A multiregional cross-sectional study
Source: PLoS One. 2024 Nov 7;19(11):e0311795. doi: 10.1371/journal.pone.0311795 (PMC11542816; doi:10.1371/journal.pone.0311795)
Supplement: Attachment — Submitted filename: Response to Reviwers 670915 for Plosone.docx [file pone.0311795.s001.docx]

**Response to reviewer**

We would like to thank you for your careful consideration of our manuscript, your pertinent questions, and your helpful suggestions. Your comments have led to several important changes to our manuscript that have considerably improved it in our opinion. We have revised our manuscript according to your recommendation, as follows:

The comments by the reviewers appear in the boxes below. The response to these comments and the associated change to the manuscript are listed below. The changes are also highlighted in the revised manuscript.

**Journal requirements:**

When submitting your revision, we need you to address these additional requirements.

1. Please ensure that your manuscript meets PLOS ONE's style requirements, including those for file naming. The PLOS ONE style templates can be found at 
<https://journals.plos.org/plosone/s/file?id=wjVg/PLOSOne_formatting_sample_main_body.pdf> and 
<https://journals.plos.org/plosone/s/file?id=ba62/PLOSOne_formatting_sample_title_authors_affiliations.pdf>

Answer:

We have revised the manuscript to ensure it adheres to PLOS ONE's style requirements.

2. Thank you for stating in your Funding Statement: 
"This study was supported by Chiang Mai University (Nuntaporn Karawekpanyawong, RG 38/2566), Thailand. The funders had no involvement in the study design, data collection, data analysis, decision to publish, or preparation of the manuscript."

Please provide an amended statement that declares *all* the funding or sources of support (whether external or internal to your organization) received during this study, as detailed online in our guide for authors at <http://journals.plos.org/plosone/s/submit-now>.  Please also include the statement “There was no additional external funding received for this study.” in your updated Funding Statement. 
Please include your amended Funding Statement within your cover letter. We will change the online submission form on your behalf.

Answer:

We have revised the funding statement as follows:

"This study was supported by Chiang Mai University (Nuntaporn Karawekpanyawong, RG 38/2566), Thailand. The funders had no involvement in the study design, data collection, data analysis, decision to publish, or preparation of the manuscript. There was no additional external funding received for this study."

**Additional Editor Comments:**

Grammar and Language
The manuscript contains a number grammatical errors and awkward sentence structures that affect readability. Inconsistent use of tense throughout the manuscript makes it difficult to follow the narrative. The flow between some sections, specifically in the introduction is sometimes poor. I would recommend a comprehensive language edit by a professional or a colleague who speaks English as a first language.

Answer:

We have thoroughly edited the manuscript to improve clarity in English usage.

Methodology
The study employs a cross-sectional design, which is inherently limited in establishing causality. This limitation should be more explicitly discussed in the methodology and discussion sections. There is no justification provided for the selection of the three specific regions of Thailand, which limits the generalisability of the findings. The statistical methods used are appropriate, but the manuscript lacks a clear explanation of how confounding variables were controlled in the regression analysis.

Answer:

- We have noted the reasons for selecting the three specific regions of Thailand due to the limitation of data in the Methods section. Please see page 5, lines 88-89.

- We have discussed the limitation of generalization of the finding due to region selection in the discussion section. Please see page 18, lines 309-311.

- We have explained how confounding variables were controlled in the regression analysis in the Methods section. Please see pages 7-8, lines 145-149.

Clarity of Results
The results section is cluttered with excessive numerical data without adequate contextual interpretation. Tables and figures are used but are not sufficiently explained in the text. A brief summary of the key points from each table should be included to aid reader comprehension.

Answer:

- We have summarized the key points from the Results section to aid reader comprehension. For Tables 1 and 2, please refer to pages 8-9, lines 156-169.
For Table 3, please refer to page 11, lines 192-198.
For Table 4, please refer to pages 12-13, lines 218-227.
For Table 5, please refer to page 14, lines 231-236.

Critical Level of the Discussion
The discussion provides a surface-level interpretation of results without delving deeply into potential reasons behind the observed patterns. There is an over-reliance on describing findings rather than critically engaging with them. Suggestions for future research are made but lack specificity and do not fully address the study's limitations. As an example, the differences in attitudes between the regions are reported, but there is little exploration of why these differences might exist beyond brief mentions in the discussion. Comparisons with existing literature are limited, reducing the critical engagement with the field. A more extensive literature integration would strengthen the discussion.

Answer:

- We have provided a deeper discussion of potential reasons behind the results, such as differences in attitudes between regions. Please refer to page 17, lines 278-291.

- We have suggested specific directions for future research. Please refer to pages 18-19, lines 322-324.

- We have fully addressed the study's limitations. Please refer to pages 18-19, lines 304-324.

**Reviewer #1:**

I would like to congratulate the authors of the article for bringing such an interesting point of view in the diversity discussion.

I think it would help the article if the authors put the "p value" in table 3, in order to help the comparisons.

Answer:

We apologize for any misunderstanding. Table 3 presents the attitudes toward LGBTQ+ physicians across six processes of medical management for all participants, without comparisons between processes or between attitudes toward non-LGBTQ+ physicians for these processes. Therefore, no p-values are provided for this result.
